# Intracranial self-stimulation mitigates spatial task deficits, modifies miR-146a and miR-495 serum levels and restores hippocampal NRF2 levels in a rat model of sporadic Alzheimer’s disease

**DOI:** 10.3389/fnagi.2025.1671196

**Published:** 2025-11-11

**Authors:** Andrea Riberas-Sánchez, Soleil García-Brito, Laia Vila-Solés, Laura Aldavert-Vera, Pilar Segura-Torres, Gemma Huguet, Gemma Carreras-Badosa, Elisabet Kádár

**Affiliations:** 1 Grup de Neurobiologia Cel·lular i Molecular, Departament de Biologia, Universitat de Girona, Girona, Spain; 2 Grup de Recerca Potenciació i Recuperació de la Memòria en Rates normals i amb Dany Cerebral, Departament de Psicobiologia i Metodologia de les Ciències de la Salut, Institut de Neurociències, Universitat Autònoma de Barcelona, Bellaterra, Barcelona, Spain

**Keywords:** Alzheimer’s disease, miRNAs, streptozotocin, intracranial self-stimulation, spatial memory

## Abstract

**Introduction:**

Alzheimer’s disease (AD) is the most prevalent neurodegenerative disease associated with aging. While deep brain stimulation (DBS) shows therapeutic promise, the long-term persistence of its effects remains understudied. Expression patterns of circulating miRNAs, proposed diagnostic biomarkers, and their modulation by DBS are still poorly characterized in longitudinal studies. This study investigates the effect of a 13-week prolonged ICSS treatment on spatial memory and serum miRNA expression in a male rat model of sporadic AD (SAD) by intracerebroventricular injection of streptozotocin (STZ).

**Methods:**

Morris water maze (MWM) tasks were conducted at 1 and 5 months post-STZ. Serum miRNA levels were quantified by qRT-PCR at 29 (Ser0), 73 (Ser1) and 136 (Ser2) days after STZ administration. Corpus callosum thickness and NRF2 protein levels in the hippocampal CA1 region were assessed using Nissl staining and immunohistochemistry, respectively. Target validation of miR-495 was performed via transfection assays in the human neuroblastoma SH-SY5Y cell line.

**Results:**

MFB-ICSS treatment significantly reduced escape latency in the MWM task in the STZ + ICSS group compared to untreated STZ rats at 5 months post-STZ. At Ser0, levels of miR-16, miR-30c, miR-181, miR-191 and miR-196a were significantly increased in STZ group. In STZ rats, miR-146a and miR-495 levels increased from Ser1 to Ser2, an effect not observed in the Control or STZ + ICSS groups. In SH-SY5Y cells, miR-495 overexpression significantly downregulated both *NRF2* mRNA and protein levels. Moreover, STZ exposure increased miR-495 and reduced NRF2 protein levels. MFB-ICSS also reversed the STZ-induced reductions in both CA1 NRF2 levels and corpus callosum thickness.

**Conclusion:**

Prolonged MFB-ICSS treatment mitigates cognitive deficits, modulates circulating levels of miRNA-495 and miR-146a, restores hippocampal NRF2 levels, and preserves corpus callosum integrity in the SAD rat model by STZ injection. These findings highlight the therapeutic potential of MFB-ICSS as a non-pharmacological intervention in AD. Furthermore, this study confirms NRF2 as a target of miR-495 in the context of AD.

## Introduction

Alzheimer’s disease (AD) is a neurodegenerative disease characterized by progressive memory loss and cognitive dysfunction, representing the main cause of dementia in elderly people. Brain pathology typically begins in medial temporal lobe areas, such as the hippocampus, and progressively extends throughout the cerebral cortex, leading to significant loss of both white and gray matter (Serrano-Pozo et al., 2011; Deture and Dickson, 2019). Currently, available pharmacological treatments for AD have a limited therapeutic effect and are unable to effectively target the widespread areas of neuronal and synaptic death observed in the AD brain (Se Thoe et al., 2021).

Most cases of AD are classified as late-onset or sporadic AD (SAD), whereas fewer than 1% of AD patients develop early-onset or familial AD. Intracerebroventricular (i.c.v) administration of streptozotocin (STZ), a toxin with diabetogenic effects in animals, is widely recognized as a suitable experimental model for SAD (Lannert and Hoyer, 1998). This model offers an alternative approach to transgenic models and i.c.v. administration of the amyloid-beta (Aβ) peptide. The STZ-induced AD model is based on the deregulation of glucose metabolism as a trigger for the disease and an inducer of the amyloid cascade and tauopathy (Hoyer, 2004). As an alkylating agent in the nitrosourea class, STZ also induces direct neuronal death through DNA methylation and DNA fragmentation (Kamat et al., 2016). Thus, *in vivo* effects of STZ may result from both processes (direct neurotoxicity and insulin-resistant brain state). It leads to progressive cognitive impairment and neuropathological alterations that closely resemble those observed in AD patients, including increased in Aβ protein, Tau hyperphosphorylation, increased oxidative stress and marked neuroinflammation and neurodegeneration (Knezovic et al., 2015; Grieb, 2016; de la Monte, 2017; Moreira-Silva et al., 2018; Amani et al., 2019; Voronkov et al., 2019).

Deep brain stimulation (DBS) has emerged as a promising therapeutic approach for AD, targeting neural circuits that underlying cognitive functions, particularly memory. Clinical studies indicate that DBS applied to regions such as the fornix, the nucleus basalis of Meynert, or the ventral capsule/ventral striatum, can delay disease progression (Lv et al., 2018). However, its long-term efficacy is unclear and may depend on the stimulation parameters, including brain targets and the duration of the DBS protocol. To date, most animal studies have employed transgenic mouse AD models using paradigms of acute DBS (e.g., single 1-h sessions) (Gallino et al., 2019) or chronic DBS (lasting no longer than 1 month) (Mann et al., 2018; Huang et al., 2019) to assess its effects on brain function and cognition. In a previous study, we demonstrated that five daily sessions of DBS targeting the medial forebrain bundle (MFB), when intracranial self-administered by experimental subjects (MFB-ICSS), improved acquisition of a spatial learning task in a male rat model of SAD induced by STZ administration (Riberas-Sánchez et al., 2024). Nevertheless, the effects of the treatment did not persist in the retention test conducted 72 h later. Recent clinical studies have suggested that alternating DBS at weekly intervals could potentiate its therapeutic effects (Peters and Tisch, 2021).

This study aims to analyze the effect of a prolonged MFB-ICSS treatment, comprising an initial intensive phase (five daily sessions) followed by an extensive phase (13 weekly sessions), on spatial learning and memory in the STZ-induced AD-like rat model.

At the molecular level, MFB-ICSS regulates hippocampal miRNAs expression levels related to AD, such as miR-495 (Puig-Parnau et al., 2020). miRNAs are small non-coding RNAs with crucial roles in gene expression regulation (Shaik et al., 2018; Sharma et al., 2024). Impaired expression of specific miRNAs has been linked to increase Aβ formation (Liu et al., 2012; Zhao et al., 2017), Tau phosphorylation (Wang et al., 2016), synaptic dysfunction (Xu et al., 2019), oxidative stress (Nunomura and Perry, 2020) and neuroinflammation (Li et al., 2024) among other pathways related to AD. Abnormal serum or plasma miRNAs expression in AD patients has also been described, and several miRNAs, including miR-16, miR-146a, miR-181a and miR-191, have been proposed as diagnostic biomarkers (Ansari et al., 2019; Varesi et al., 2022). However, the analysis of circulating miRNAs levels as biomarkers for monitoring disease progression and evaluating the effectiveness of potential AD treatments has been poorly studied. Longitudinal follow-up studies in animal models of AD are therefore required to validate the therapeutic effects of DBS and its impact on the time course of the miRNAs profile as potential biomarkers for future clinical applications.

The related pathways of many of the miRNAs significantly altered in AD are not yet fully delineated, and most of their putative targets remain unvalidated. This is the case of miR-495, which has been reported to be downregulated in the gray matter of the temporal cortex in early-stage AD patients (Wang et al., 2011). No studies to date have examined its circulating expression levels and its specific role in the context of AD remains poorly understood.

This study also aims to analyze the effect of the aforementioned MFB-ICSS treatment on serum expression levels of a subset of AD-related miRNAs, including miR-495, throughout disease progression. It assesses the impact of the treatment on the temporal evolution of these miRNA levels. Additionally, putative targets of miR-495 are evaluated in the human neuroblastoma SH-SY5Y cell line. The effects of MFB-ICSS treatment on corpus callosum thickness, as a structural biomarker, and the nuclear factor erythroid 2-related factor 2 (NRF2) levels, as a key regulator of antioxidant response and a target of miR-495, are also analyzed in the hippocampal CA1 region of STZ rats.

## Materials and methods

### Animals

Adult male Wistar rats (Harlan Laboratories, Horst, The Netherlands) between 13 and 14 weeks old and with an average weight of 403.5 g (± 18.01) at the time of surgery were used for this study. The study was approved by the University Animal Welfare Committee (CEEAH, protocol number 4848 P1) and was in compliance with the European Community Council Directive (86/609/CEE, 92/65/CEE, 2010/63/UE), Royal Decree 53/201. Rats were individually housed in a controlled environment (21 ± 1 °C; humidity, 60%; lights on from 8:00 a.m. to 8:00 p.m.; food and water available *ad libitum*).

### Experimental groups

**Figure 1:**
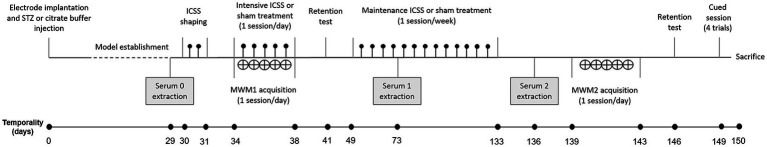
Experimental design timeline. On day 0, rats underwent stereotactic surgery to inject intracerebroventricular streptozotocin (STZ) or citrate buffer (control) and to implant a monopolar electrode at the MFB. Subjects were trained on the Morris Water Maze (MWM) task between day 34 and 38 (MWM1) and again between day 139 and 143 (MWM2) post-STZ injection, with a retention test applied on days 41 and 146, respectively. A cued session was conducted on day 149. The STZ+ICSS group received a prolonged MFB-ICSS treatment protocol, including an initial intensive treatment (1 session/day during 5 consecutive days), contingent with MWM, followed by a non-training-contingent extensive MFB-ICSS administration (1 session/week during 13 weeks). Control and STZ groups received sham treatment.

A total of 27 rats underwent a stereotaxic procedure for intracerebroventricular injection and electrode implantation. The animals were divided into two groups: the vehicle injected control group (Control group, *n* = 11) and the STZ-injected group (STZ, *n* = 16). On day 34, the STZ-injected rats were further divided into two groups: those receiving ICSS treatment (STZ + ICSS group, *n* = 8) and those receiving sham treatment (STZ group, *n* = 8). Behavioral training in the Morris Water Maze (MWM) was conducted between days 34 and 41 (MWM1) and again between days 139 and 143 (MWM2). The Control group underwent the same behavioral tests in the MWM and sham treatment as the STZ rats. The experimental timeline is shown in Figure 1.

### Intracerebroventricular injection of STZ and electrode implantation

Animals were anesthetized with a mixture of 5% isoflurane and oxygen and subjected to stereotactic surgery. As described in our previous study (Riberas-Sánchez et al., 2024), 8 μL of STZ (2 mg/kg) (Merck Life Science, catalog number S0130-50MG) or vehicle (citrate buffer) were bilaterally injected into the lateral ventricles at a rate of 0.7 μL/min, at DV = −4.00 mm, with the cranial surface as the dorsal reference. Following the i.c.v. injection, a monopolar stainless steel electrode (150 μm in diameter) was unilaterally and chronically implanted into the MFB of the right lateral hypothalamus (AP = −2.3 mm; L = −1.8 mm; DV = −8.8 mm) (Vila-Solés et al., 2022).

### Intracranial self-stimulation treatment

Electrical brain stimulation consisted of 0.3-s trains of 50 Hz sinusoidal waves (40–175 μA). For each subject, the optimum current intensity (OI) was established as the mean of the two current intensities that produced the highest response rate in the two shaping sessions (responses/min) (see Vila-Soles et al. for details) (Vila-Solés et al., 2022). Rats in the STZ + ICSS group underwent intracranial self-stimulation (ICSS) treatment, consisting of an initial intensive phase (five post-training daily sessions contingent on each of the acquisition sessions in the MWM), followed by 13 non-training-contingent weekly sessions. In total, the MFB-ICSS treatment lasted 4 months. In each ICSS treatment session, rats were placed in a conventional Skinner box and allowed to press a lever to self-administer 2,500 trains of electrical stimulation at their individualized OI. Rats in the STZ and Control groups were handled and placed in the Skinner box for 30 min, without receiving electrical stimulation (sham treatment).

### Morris water maze

The MWM task was used to assess spatial learning and memory at the two previously described time points: approximately 1 month (MWM1) and 5 months (MWM2) after STZ administration. Both MWM1 and MWM2 consisted of 5 daily acquisition sessions (A1–A5), each consisting of 4 trials with a mean intertrial interval of 120 s. Seventy-two hours after the last acquisition session in each phase, animals completed a retention test (on days 41 and 146), which involved removing the platform and allowing the animals to swim for 60 s, starting from the East position. In addition, a 4-trial session following a cued procedure was conducted 3 days after the final retention test (day 149) to control for non-spatial factors that could affect the animal’s performance in the task, such as visual and motor abilities as well as their motivation to mount the platform. The principal quantitative outcome measures for acquisition and cued sessions were escape *latency* or time (seconds) required to locate and mount the platform. For the retention test, the percentage of time spent in the *target quadrant (*TQ) and in *the target annulus* (TA), the *mean distance* to target (MDT), and the *Whishaw’s Error* (WE) were analyzed. Additional control variables included the percentage of time spent near the *walls* (Walls), as a measure of thigmotaxis–anxiety, and the *swimming speed* across each trial and session.

Swim paths were recorded using a closed-circuit video camera and the Smart Video Tracking System (Version 3.0, Panlab). Swimming trajectories were analyzed during the fifth acquisition session of both MWM1 and MWM2. For each animal, the predominant search strategy across the four trials of the session was classified according to categorization by Rogers et al. (2017), as Non-spatial (Random swimming, Thigmotaxis), Semi-spatial (Scanning, Circling), or Spatial (Chaining, Goal-directed search, Direct swimming).

### Animal sample collection

Serum samples were collected from all rats on days 29 (Ser0), 73 (Ser1) and 136 (Ser2) via lateral tail vein extraction, using Microvette tubes (Microvette® CB 300, SARSTEDT Sau). The tubes were kept at room temperature for 45 min and centrifuged at 3,000 rpm for 10 min to collect supernatant and obtain serum. Serum samples were stored at −80 °C until the isolation of total RNA using the mirVana PARIS Kit (Invitrogen™, ThermoFisher Scientific™). Tissue samples were obtained after the animals’ sacrifice with pentobarbital (200 mg/kg body weight, i.p.) on day 150. Ipsilateral brain hemispheres were fixed in 4% formaldehyde solution in phosphate buffered saline (PBS) for 24 h at 4 °C, post-fixed for 48 h and cryopreserved in 15 and 30% sucrose PBS solution subsequently. Finally, coronal sections of 35 μm and 12 μm thickness were obtained using a cryostat and stored at −80 °C until their use.

### *In vitro* assessments in the human neuroblastoma SH-SY5Y cell line

The human neuroblastoma SH-SY5Y cell line was purchased from the American Type Culture Collection (ATCC, CRL-2266, deposited by JL Biedler) and cultured in DMEM/F12 GlutaMAX™ (Dulbecco’s Modified Eagle Medium: Nutrient Mixture F12, Gibco™, ThermoFisher Scientific™) supplemented with 10% of heat-inactivated fetal serum bovine (FBS) (Gibco™, ThermoFisher Scientific™) and 1% penicillin–streptomycin (P/S) (Corning®, Cultek) in a humidified atmosphere containing 5% CO2 at 37 °C following manufacturer instructions.

For transfection experiments, SH-SY5Y cells were seeded in a 6-well plate (1×10^6 cells/well) and transfected with 30 nM of the miRNA-495 mimic (MC11526), miRNA-495 inhibitor (MH11526), the negative control mimic NEG-1 (NC mimic) (4464058) or negative control inhibitor (NC inhibitor) (4464076) (Invitrogen™, ThermoFisher Scientific™) using Lipofectamine™ RNAiMAX (Invitrogen™, ThermoFisher Scientific™) for 48 h according to the Forward protocol described by Invitrogen™. Each condition was tested in triplicate in three independent experiments.

For STZ administration, SH-SY5Y cells were seeded into 6-well plates and treated with 1 mM of STZ (Sigma-Aldrich, Merck) or vehicle (citrate buffer) during 24 h according to the protocol described by Plaschke et al. for human neuroblastoma cells (Plaschke and Kopitz, 2015). Each condition was tested in triplicate in three independent experiments.

Total RNA and protein from the SH-SY5Y cells were isolated using the mirVana PARIS Kit (Invitrogen™, ThermoFisher Scientific™), previous mechanical detachment using a scraper and 200 μL of Cell Disruption Buffer. RNA and protein samples were quantified using a NanoDrop 1,000 Spectrophotometer (ThermoFisher Scientific™) or Pierce BCA Protein Assay kit (ThermoFisher Scientific™) and a Synergy 4 spectrophotometer (BioTek), respectively.

### TaqMan miRNA qRT-PCR

From total RNAs, cDNAs were synthetized and preamplified with the TaqMan® Advanced miRNA cDNA Synthesis Kit (Applied Biosystems) in an AB Veriti 96-well thermocycler (Applied Biosystems). PCRs were run on an AB QuantStudio 7, using the TaqMan Advanced miRNA qPCR assays rno-miR-16-5p, hsa-miR-30-5p, rno-miR-132-3p, rno-miR-146a-5p, hsa-miR-181a-5p, rno-miR-181c-5p, hsa-miR-191-5p, hsa-miR-196a-5p and mmu-miR-495-3p or hsa-miR-495-3p (Applied Biosystems). Based on the most stable endogenous candidate identified by the NormFinder algorithm, hsa-miR-let-7a-5p (Applied Biosystems) was used as the normalizer to estimate the relative quantity of each target miRNA, which was calculated as 2^^−^ΔΔCт (ΔΔCт = ΔCt sample − ΔCt reference sample; ΔCt = Ct target − Ct normalizer). The mean level of Ser 0 in the Control group was used as the reference sample and set to 1 in the serum sample. In cell samples, the mean level in the Control group was used as the reference sample.

### TaqMan mRNA qRT-PCR

Ten μL of RNA from cell extracts, treated with TURBO DNA-free (ThermoFisher Scientific™), were used for cDNA synthesis with the High Capacity cDNA Reverse Transcription Kit (Applied Biosystems™, ThermoFisher Scientific™) and an AB Veriti 96 well thermocycler (Applied Biosystems™, ThermoFisher Scientific™). Quantitative reverse transcription PCR (qRT-PCR) was run on an AB QuantStudio 7, using the TaqMan Gene Expression Assays (Fatty acid elongase 7) (*ELOVL7*) (Hs00405151_m1); *NRF2* (Hs00232352_m1); insulin like growth factor 1 receptor (*IGF1R*) (Hs00609566_m1); Tumor necrosis factor receptor superfamily, member 1B (*TNFRSF1B*) (Hs00961750_m1) and hypoxanthine phosphoribosyltransferase 1 (*HPRT1*) (Hs02800695_m1) (Applied Biosystems™, ThermoFisher Scientific™). The relative quantification of each mRNA was determined as 2^−ΔΔCт^, where ΔΔCт it is calculated as ΔCt sample − ΔCt reference sample and ΔCt is defined as Ct target − Ct normalizer. The mean of the Control group was used as a reference sample, and *HPRT* mRNA levels served as endogenous normalizer.

### Western blot

A total of 15 μg of protein was loaded onto Criterion TGX Stain-Free PreCast Gels with 18-well comb (Bio-Rad) under reducing conditions, and subsequently electrotransferred to PVDF membranes. After 1 h of blocking with 5% Bovine Serum Albumin (Sigma-Aldrich, Merck) in TBS-T (tris-buffered saline [100mMNaCl, 10mMTris-HCl pH 7.5] containing 0.1% Tween-20), membrane was incubated with the primary antibody NRF2 (1:500, sc-365949, Santa Cruz Biotechnology) at 4 °C overnight. Membranes were then incubated for 1 h at room temperature with a peroxidase-conjugated goat anti-mouse secondary antibody (1:20,000, no. 115–035-044, Jackson ImmunoResearch). Intensities of antibody reactive bands were detected using Immobilon Western Chemiluminescent HRP Substrate (EMD Millipore) in a FluorChem luminometer, and quantified by densitometry using FluorChem SP software (AlphaEaseFC™). Protein relative intensities were normalized by total protein lanes as loading controls according to Aldridge et al. (2008).

### Nissl staining

Histological sections (12 μm thick) were stained using a filtered 0.5% cresyl violet solution for 3.5 min, rinsed and washed in distilled water for 5 min, and subsequently dehydrated and cover-slipped in Entellan mounting medium (Sigma-Aldrich, Merck). Photomicrographs of the corpus callosum region between Bregma coordinates −3.4 to −3.6 were acquired with a Vanox-T AH-2 microscope (Olympus). Quantification of corpus callosum thickness was performed in a blinded manner by two independent investigators using CellSens Standard imaging software. For each subject, corpus callosum thickness was calculated as the mean of the values obtained from three histological sections.

### NRF2 immunostaining

To assess NRF2 levels in the CA1 region of the hippocampus, free-floating immunohistochemistry was performed. Coronal sections of 35 μm in thickness were placed in 12-well plates containing Tris Buffer Saline (TBS) and fixed with 2% formaldehyde TBS for 20 min at room temperature (RT). Samples were incubated in 0.1% H_2_O_2_ TBS for 15 min followed by 7% Normal Goat Serum for 1 h at RT. Sections were then incubated with primary antibody anti-NRF2 (PA5-27882, Invitrogen™, ThermoFisher Scientific™) diluted 1:500 in TBS-T 1% BSA for 48 h. After washing with TBS-T, sections were incubated with a biotinylated secondary antibody (Goat anti-rabbit IgG, 111–066-144, Jackson ImmunoResearch) diluted 1:800 in TBS-T 1% BSA for 1.5 h under the same conditions. Subsequently, samples were incubated with ABC solution (Vectastain Elite ABC-Peroxidase kit, PK-6100, Vector Laboratories) for 40 min at RT followed by a 2-min incubation with DAB. Finally, sections were mounted onto gelatin-coated slides, dehydrated and cover-slipped with Histomount mounting medium (HS-103, National Diagnostics).

Photomicrographs of the hippocampal CA1 region between Bregma coordinates −3.2 to −3.8 were acquired using an Olympus Vanox-T AH-2 microscope. NRF2 immunoreactivity was quantified using Fiji (ImageJ, NIH), by measuring the mean gray value within circular ROIs manually placed over the pyramidal layer by two independent investigators in a blinded manner. For each animal, three ROIs were analyzed per section across three hippocampal sections.

### Statistical analyses

Statistical analyses were performed using IBM SPSS Statistics 25. Performance in the MWM was analyzed using a 3 × 5 two-way mixed ANOVA (GROUP × SESSION) for the acquisition sessions, with the average score of four trials per session. Variables in the retention test, cued session (average score of the four trials), serum miRNAs and hippocampal CA1 NRF2 expression levels were analyzed using one-way ANOVA for parametric data or the Kruskal–Wallis test for nonparametric data (according to normality analysis results), followed by the appropriate *post-hoc* tests. A one-sample *t*-test against a constant was used to determine whether the percentage of time spent in the TQ differed from chance (25%) for each group. The distribution of swimming strategies was analyzed using the Chi-square test to assess group differences in categorical data. Serum miRNAs levels were longitudinally compared across the three time-points (Ser0, Ser1 and Ser2) and the experimental groups using a 3 × 3 two-way mixed ANOVA (GROUP × TIME). NRF2 expression levels in transfected SH-SY5Y cells were compared to the respective controls using an independent samples *t*-test, for parametric comparisons, or a Mann–Whitney U test, for nonparametric comparisons. Correlations between variables were assessed using Spearman’s bivariate correlation test. Results were considered to be statistically significant at *p* < 0.05, with a 95% confidence interval. The rank-biserial correlation (*r*), Hedges’s g (*g*), partial Eta squared (η_p_^2^), Epsilon squared (ε^2^) or Cohen’s d (*d*) were used as effect size estimates according to the used test.

## Results

### MFB-ICSS treatment improved performance on the spatial MWM task 5 months after STZ infusion

We analyzed the acquisition and retention of the spatial MWM task in a SAD-like rat model at two time points following STZ administration: 1 month (MWM1) and 5 months (MWM2). In MWM1, we also assessed the effects of ICSS administered post-training during the acquisition sessions (5 sessions), while MWM2 was conducted after completing 13 additional ICSS sessions, administered once per week and non-contingent on training.

**Figure 2:**
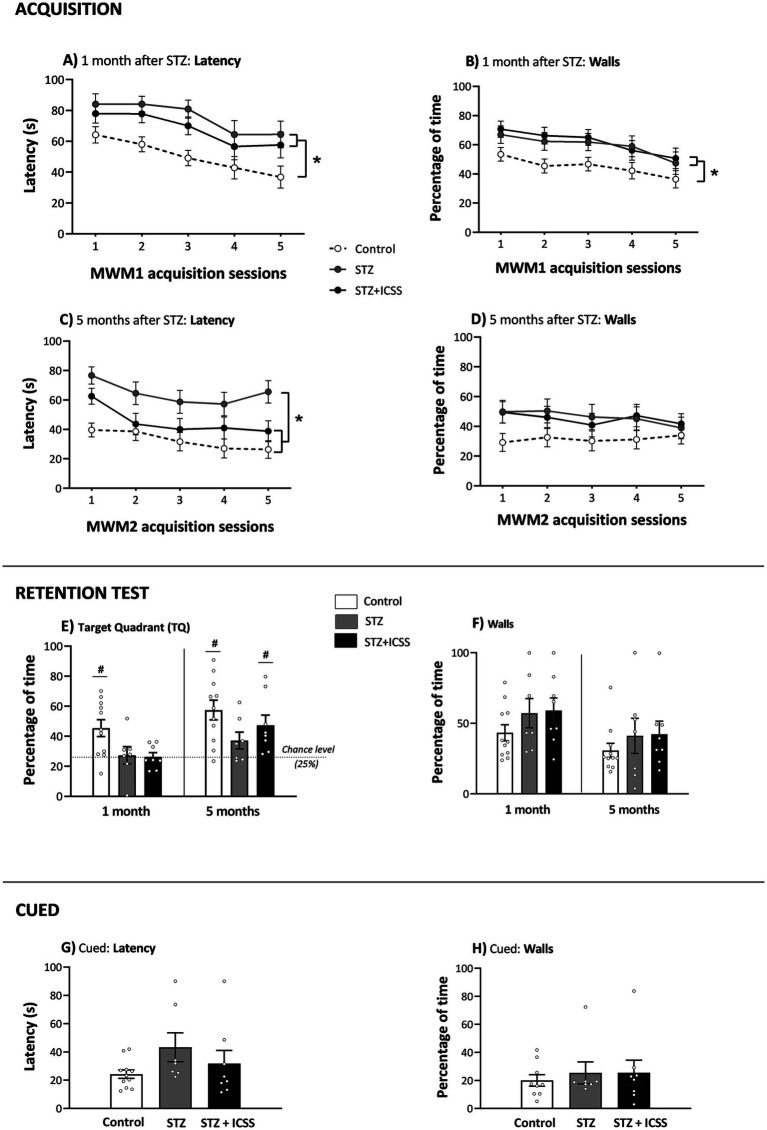
Effects of STZ and MFB-ICSS on spatial task performance in the Morris Water Maze at 1 and 5 months after STZ administration. Escape latencies for the 5 acquisition sessions and percentage of time near walls during the MWM1 and MWM2 for Control, STZ and STZ+ICSS groups after 1 **(A,B)** and 5 **(C,D)** months post-STZ, respectively. *p < 0.05 relative to the Control group. Percentage of time spent in the target quadrant **(E)** and percentage of time near walls **(F)** in the retention tests at 1 month and 5 months post-STZ. Dotted line in **(E)** represents chance level (25%) and significant differences for each group and chance level are depicted with *p < 0.05. Latency **(G)** and percentage of time near walls **(H)** in the cued session at 5 months post-STZ. Data are presented as mean ± SEM.

As shown in Figure 2A, the acquisition results in MWM1 revealed significant differences between the three experimental groups (GROUP: *F*_2,23_ = 8.01; *p* = 0.002, η_p_^2^ = 0.41), regardless of the session (GROUP × SESSION: *F*_8,92_ = 0.28; *p* = 0.97, η_p_^2^ = 0.02). STZ rats, independent of ICSS treatment, displayed an impaired learning curve compared to Control rats (STZ: *p* = 0.001; STZ+ICSS: *p* = 0.01). No significant differences were detected between the STZ and STZ+ICSS groups (*p* = 0.30). However, intra-group analysis revealed that only the STZ group did not show a clear significant reduction in escape latencies between the first and the last training session (Control: *p* = 0.02; STZ: *p* = 0.08; STZ+ICSS: *p* = 0.03). Regarding the time spent near the walls, although all groups exhibited a linear downward trend over the 5 sessions (*F*_1,23_ = 15.96; *p* = 0.001, η_p_^2^ = 0.41; GROUP × SESSION *F*_2,23_ = 0.13; *p* = 0.88, η_p_^2^ = 0.01), there were differences between the groups (*F*_2,23_ = 4.47; *p* = 0.02, η_p_^2^ = 0.28), with both STZ groups spending more time near the walls compared to the Control group (STZ: *p* = 0.03; STZ+ICSS: *p* = 0.01) (Figure 2B).

In the probe test conducted 72 h after the MWM1 last acquisition session, differences between groups were observed in the TQ (*F*_2,25_ = 4.88, *p* = 0.02, η_p_^2^ = 0.30), TA (*F*_2,25_ = 5.80, *p* = 0.009, η_p_^2^ = 0.34) and MDT (*F*_2,25_ = 3.83, *p* = 0.04, η_p_^2^ = 0.25). Specifically, both STZ groups spent less time in the target quadrant (STZ: *p* = 0.02; STZ+ICSS: *p* = 0.01) and in the target annulus (STZ: *p* = 0.01; STZ+ICSS: *p* = 0.006), and had a greater mean distance to the target (STZ: *p* = 0.05; STZ+ICSS: *p* = 0.02) compared to the Control group. In the TQ test, only the Control group performed above the level of chance (25%) (Control: *t_10_* = 3.69, *p* = 0.004; STZ: *t_6_* = 0.41, *p* = 0.7; STZ+ICSS: *t_7_* = 0.51, *p* = 0.63, *t*-test) (Figure 2E). No significant differences were observed between groups in either the EW or the Walls variables (Figure 2F).

At 5 months, the group factor was found to be significant again (*F*_2,23_ = 8.30, *p* = 0.002, η_p_^2^ = 0.42), regardless of session (GROUP × SESSION: *F*_8,92_ = 0.97, *p* = 0.46, η_p_^2^ = 0.08) (Figure 2C) during the acquisition phase. STZ animals had significantly longer latencies than those in the Control group (*p* < 0.001). In contrast to the findings in MWM1, the STZ+ICSS group, after 13 additional ICSS sessions, performed better than the STZ group (*p* = 0.03). Intra-group analysis of latency progression across acquisition sessions showed that, once again, the STZ group does not improve its performance between the first and the last session (*p* = 0.13), whereas the Control (*p* = 0.02) and STZ+ICSS groups (*p* = 0.002) did. Regarding the time spent near the walls, all groups displayed similar durations, which remained consistent throughout the sessions (SESSION: *F*_4,92_ = 1.00, *p* = 0.41, η_p_^2^ = 0.04; GROUP × SESSION: *F*_8,92_ = 0.93, *p* = 0.50 η_p_^2^ = 0.07; GROUP: *F*_2,23_ = 1.85, *p* = 0.18, η_p_^2^ = 0.14) (Figure 2D).

In the MWM2 retention, STZ rats continued to show poorer retention 72 h after the last acquisition session compared to Control rats. Group differences in retention test variables trended toward significance (TQ: *F*_2,25_ = 2.38, *p* = 0.12, η_p_^2^ = 0.17; TA: *F*_2,25_ = 2.63, *p* = 0.09, η_p_^2^ = 0.19; EW: *F*_2,25_ = 3.10, *p* = 0.07, η_p_^2^ = 0.23), the STZ group spent less time in the TQ (*p* = 0.04) and had a lower WE (*p* = 0.03) compared to the Control group. Nevertheless, the STZ+ICSS group did not differ from the Control group. The Control and the STZ+ICSS groups performed above chance level (25%) in the TQ (Control: *t_10_* = 4.95, *p* = 0.001; STZ: *t_6_* = 2.16, *p* = 0.07; STZ+ICSS: *t_7_* = 3.30, *p* = 0.01) (Figure 2E). No differences were observed between groups in the time spent near the walls during this test (Figure 2F).

In the Cued session, no differences were observed between groups in either swim latencies (Figure 2G) or time spent near the walls (Figure 2H). Finally, no differences in swim speed were observed in any session, either during the spatial protocol or the Cued session.

**Figure 3:**
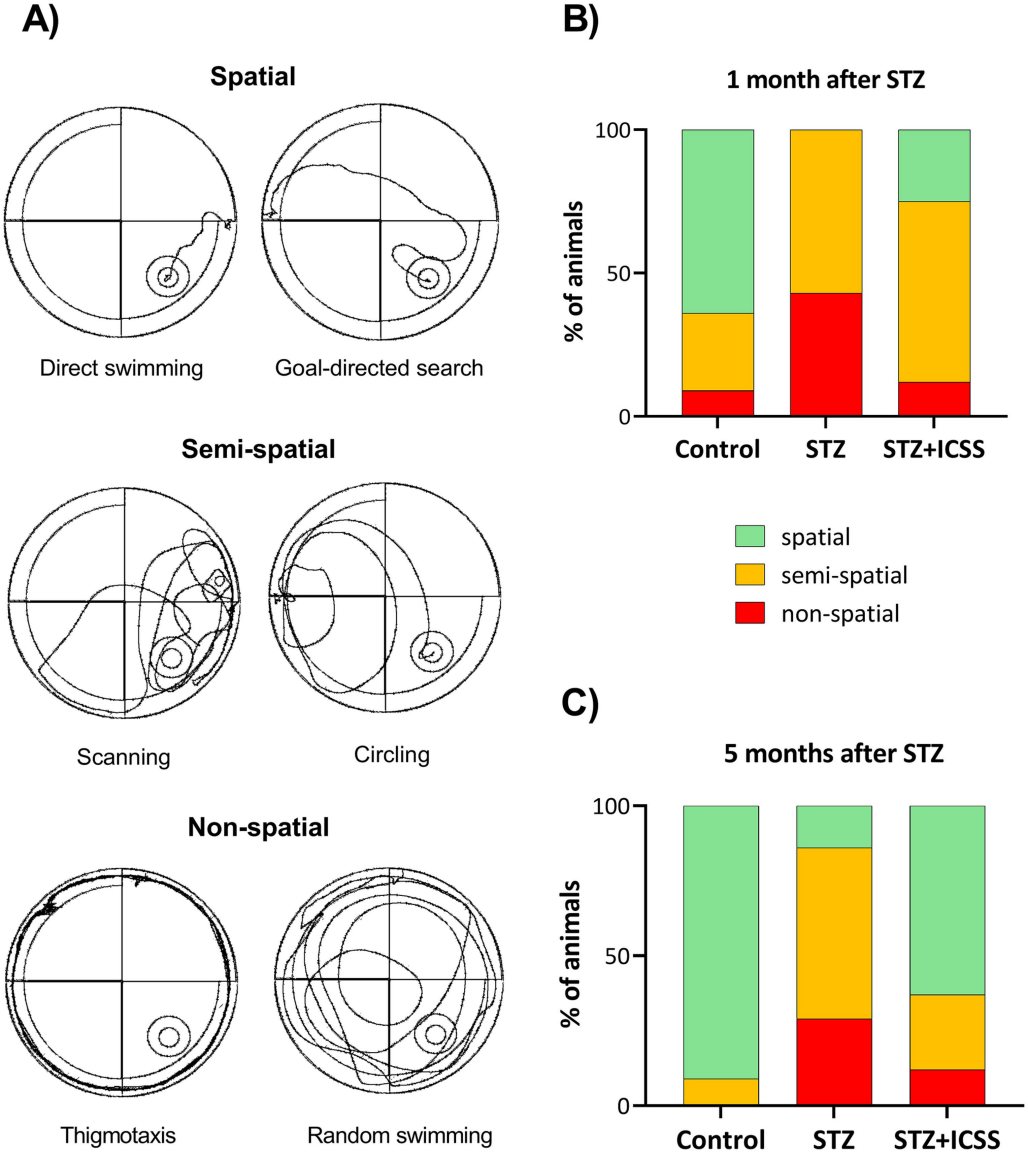
Swim strategies in the Morris Water Maze. Representative images of the swimming strategies classified as spatial, semi-spatial, and non-spatial **(A)**. Predominant swim trajectories adopted by the three experimental groups—Control, STZ, and STZ+ICSS—during the fifth acquisition session of the Morris Water Maze at 1 month **(B)** and 5 months **(C)** after STZ. Data are presented as the percentage of animals exhibiting each strategy.

As shown in Figure 3, trajectory analysis revealed differences between groups both at 1 and 5 months post-STZ administration (χ^2^₄ = 9.618, *p* = 0.047; χ^2^_4_ = 10.803, *p* = 0.029, respectively). The STZ rats predominantly exhibited non-spatial or semi-spatial exploratory behaviors, whereas control rats consistently relied on hippocampal-dependent spatial strategies. In STZ rats receiving ICSS, a shift was observed from predominantly semi-spatial strategies in MWM1 to spatial strategies in MWM2.

### Serum miRNA levels were altered at 29 days after STZ administration

**Figure 4:**
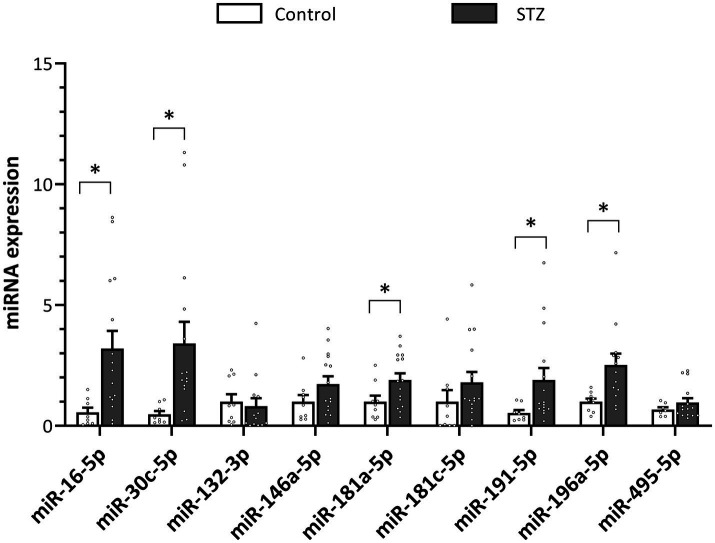
Effect of STZ on miRNA expression serum levels in rats 29 days (Ser0) after injection. Relative quantity of each target miRNA was determined as ^2^(−ΔΔCt) (ΔΔCт = ΔCt sample − ΔCt reference sample; ΔCt = Ct target − Ct normalizer), using the mean level of the Control group as the reference sample and miR-let-7a-5p as normalizer. Data are presented as mean ± SEM. *p < 0.05 relative to the Control group.

We analyzed the serum expression of 9 miRNAs related to neural plasticity, postulated as potential biomarkers of AD, at different times post-STZ or vehicle administration. As shown in Figure 4, levels of miR-16, miR-30c, miR-181a, miR-191 and miR-196a were significantly upregulated in all STZ rats (both STZ or STZ+ICSS groups, as ICSS treatment had not yet been applied) compared to the Control group (*U* = 19, *p* = 0.007, *r* =0.55; *U* =13, *p* = 0.001, *r* = 0.63; *t_22_* = 2.15, *p* = 0.04, *g* = 0.88; *U* = 25, *p* = 0.02, *r* = 0.47; *U* = 16, *p* = 0.003, *r* = 0.61, respectively) at 29 days post-STZ (Ser0). However, no significant changes on the levels of miR-132, miR-146a, miR-181c and miR-495 were observed between groups at 29 days post-STZ.

No significant differences in serum miRNAs levels were observed among the experimental groups at 73 days (Ser1) or 136 days (Ser2) post-STZ (Supplementary Figure S1).

### Serum miRNA levels at 29 days after STZ administration were associated with worse spatial memory performance in the MWM task 5 months after STZ administration

**Figure 5:**
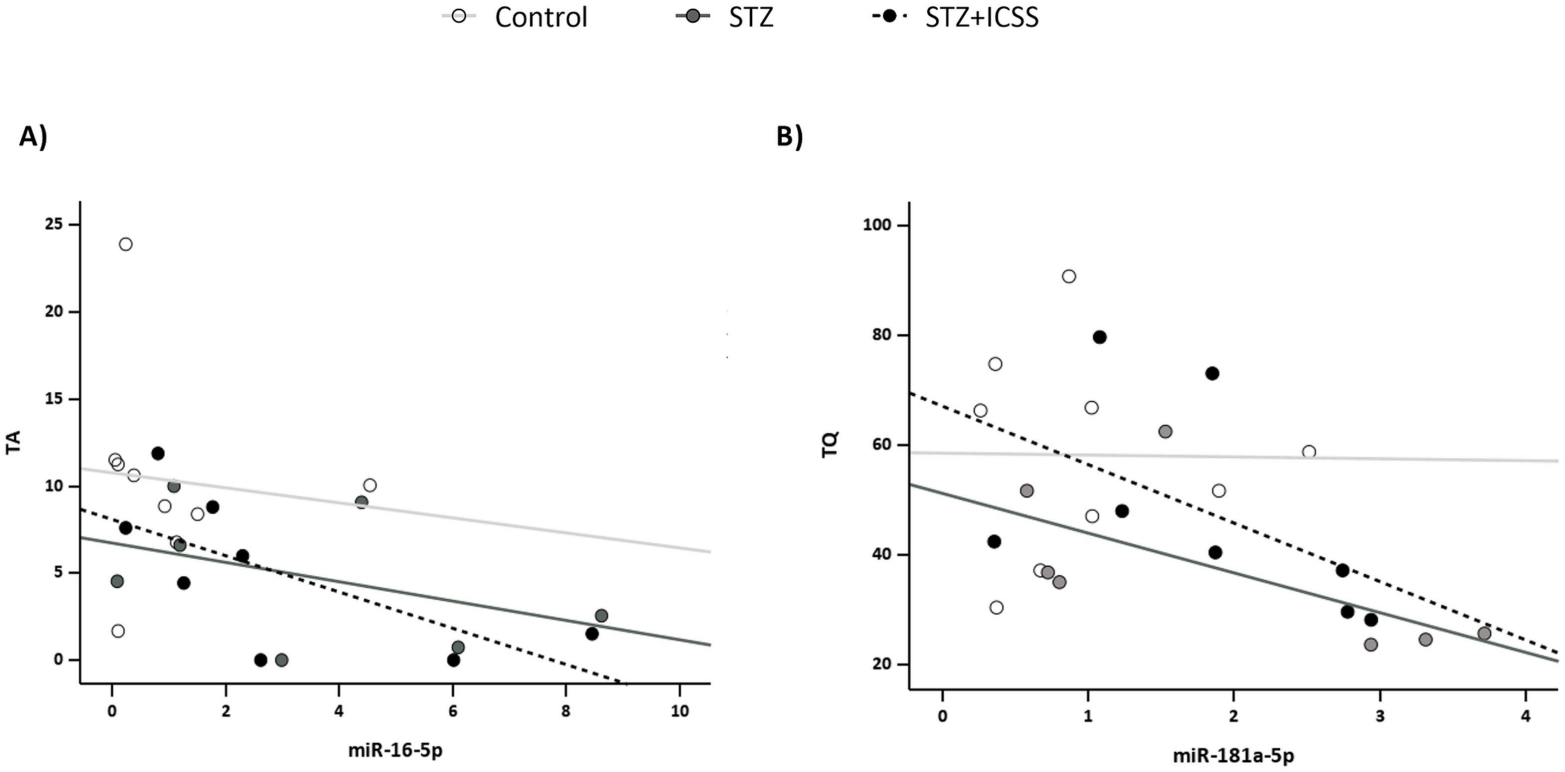
Correlations between Ser0 miR-16 and miR-181a and MWM retention test variables at 5 months after STZ administration. Correlation between Ser0 miR-16 and the percentage of time spent in the target annulus (TA) **(A)** and correlation between Ser0 miR-181a and the percentage of time spent in target quadrant (TQ) **(B)**. Each dot represents an individual rat, and the linear trend for each Control, STZ or STZ+ICSS group is shown.

The correlation analysis between modulated serum miRNAs expression levels at 29 days after STZ administration, and behavioral performance in MWM2 for the experimental groups is summarized in Table 1. Ser0 levels of miR-16, miR-30c, miR-181a, and miR-191 and miR-196a were significantly associated with worse performance in MWM2 in the STZ+ICSS group. Specifically, Ser0 levels of these miRNAs positively correlated with latencies during acquisition sessions (all *ρ* > 0.7). Negative correlations were also observed between Ser0 levels of miR-16 and miR-181a, and the TA and TQ variables from the retention test of MWM2 (Figure 5). miR-181a negatively correlated with WE, while miR-16, miR-181a and miR-191 positively correlated with MDT. In the STZ group, Ser0 levels of miR-196a correlated with the TA variable. No correlations were observed in the Control group.

**Table 1:** Correlation analyses between miR-16, miR-30c, miR-181a, miR-191 and miR-196a serum levels at 29 days after STZ injection and behavioral variables in Morris Water Maze 2.

miRNA	Ser 0	A1	A2	A3	A4	A5	TQ	TA	MDT	WE
Control group										
miR-16	Correlation coefficient	0.367	0.467	0.250	−0.383	0.050	−0.167	−0.450	0.300	0.350
	Sig. (2-tailed)	0.332	0.205	0.516	0.308	0.898	0.668	0.224	0.433	0.356
	*N*	9	9	9	9	9	9	9	9	9
miR-30c	Correlation coefficient	0.183	0.267	0.183	−0.450	0.067	−0.033	−0.200	0.150	0.317
	Sig. (2-tailed)	0.637	0.488	0.637	0.224	0.865	0.932	0.606	0.700	0.406
	*N*	9	9	9	9	9	9	9	9	9
miR-181a	Correlation coefficient	0.350	0.500	0.300	−0.433	0.150	−0.117	−0.367	0.267	0.500
	Sig. (2-tailed)	0.356	0.170	0.433	0.244	0.700	0.765	0.332	0.488	0.170
	*N*	9	9	9	9	9	9	9	9	9
miR-191	Correlation coefficient	0.317	0.400	0.067	−0.133	−0.050	0.167	−0.233	−0.017	0.333
	Sig. (2-tailed)	0.406	0.286	0.865	0.732	0.898	0.668	0.546	0.966	0.381
	*N*	9	9	9	9	9	9	9	9	9
miR-196a	Correlation coefficient	−0.333	−0.150	−0.300	0.050	−0.367	0.617	0.200	−0.500	−0.017
	Sig. (2-tailed)	0.381	0.700	0.433	0.898	0.332	0.077	0.606	0.170	0.966
	*N*	9	9	9	9	9	9	9	9	9
STZ group										
miR-16	Correlation coefficient	−0.143	0.000	0.429	0.286	0.500	−0.429	−0.429	0.464	−0.607
	Sig. (2-tailed)	0.760	1.000	0.337	0.535	0.253	0.337	0.377	0.297	0.148
	*N*	7	7	7	7	7	7	7	7	7
miR-30c	Correlation coefficient	−0.143	0.000	0.429	0.286	0.500	−0.429	−0.429	0.464	−0.607
	Sig. (2-tailed)	0.760	1.000	0.337	0.535	0.253	0.337	0.377	0.297	0.148
	*N*	7	7	7	7	7	7	7	7	7
miR-181a	Correlation coefficient	0.071	0.179	0.643	0.393	0.643	−0.643	−0.607	0.679	−0.643
	Sig. (2-tailed)	0.879	0.702	0.119	0.383	0.119	0.119	0.148	0.094	0.119
	*N*	7	7	7	7	7	7	7	7	7
miR-191	Correlation coefficient	−0.143	0.000	0.429	0.286	0.500	−0.429	−0.429	0.464	−0.607
	Sig. (2-tailed)	0.760	1.000	0.337	0.535	0.253	0.337	0.337	0.294	0.148
	*N*	7	7	7	7	7	7	7	7	7
miR-196a	Correlation coefficient	0.029	0.086	−0.600	−0.543	−0.657	0.600	0.886*	−0.429	0.600
	Sig. (2-tailed)	0.957	0.872	0.208	0.266	0.156	0.208	**0.019**	0.397	0.208
	*N*	6	6	6	6	6	6	6	6	6
STZ+ICSS group										
miR-16	Correlation coefficient	0.755*	0.333	0.524	0.762*	0.643	−0.810*	−0.755*	0.762*	−0.643
	Sig. (2-tailed)	**0.031**	0.420	0.183	**0.028**	0.086	**0.015**	**0.031**	**0.028**	0.086
	*N*	8	8	8	8	8	8	8	8	8
miR-30c	Correlation coefficient	0.395	0.429	0.714*	0.476	0.405	−0.452	−0.611	0.500	−0.190
	Sig. (2-tailed)	0.333	0.289	**0.047**	0.233	0.320	0.260	0.108	0.207	0.651
	*N*	8	8	8	8	8	8	8	8	8
miR-181a	Correlation coefficient	0.611	0.071	0.405	0.667	0.714*	−0.833*	−0.874**	0.714*	−0.714*
	Sig. (2-tailed)	0.108	0.867	0.320	0.071	**0.047**	**0.010**	**0.005**	**0.047**	**0.047**
	*N*	8	8	8	8	8	8	8	8	8
miR-191	Correlation coefficient	0.491	0.405	0.738*	0.405	0.310	−0.667	−0.659	0.714*	−0.381
	Sig. (2-tailed)	0.217	0.320	**0.037**	0.320	0.456	0.071	0.076	**0.047**	0.352
	*N*	8	8	8	8	8	8	8	8	8
miR-196a	Correlation coefficient	0.395	0.048	0.714*	0.381	0.167	−0.429	−0.323	0.524	−0.167
	Sig. (2-tailed)	0.333	0.911	**0.047**	0.352	0.693	0.289	0.435	0.183	0.693
	*N*	8	8	8	8	8	8	8	8	8

A: Acquisition session (A1-A5); TQ: Target quadrant; TA: Target annulus; MDT: Mean distance to target; WE: Whishaw’s Error. **Correlation is significant at the 0.01 level (2-tailed). *Correlation is significant at the 0.05 level (2-tailed). The table includes all the correlations found in Control, STZ and STZ+ICSS groups according to Spearman’s correlation test (*p* < 0.05, significant correlations are highlighted in the table). Significant correlations have been highlighted in bold and grey box in the table.

Correlations between Ser1 or Ser2 miRNA levels and behavioral performance during acquisition or retention of MWM2 are summarized in Tables 2, 3.

**Table 2:** Correlation analyses between miR-16, miR-30c, miR-181a, miR-191 and miR-196a serum levels at 73 days after STZ injection and behavioral variables in Morris Water Maze 2.

miRNA	Ser 1	A1	A2	A3	A4	A5	TQ	TA	MDT	WE
Control group										
miR-16	Correlation coefficient	−0.267	−0.117	−0.167	0.600	−0.250	0.600	0.367	−0.583	−0.033
	Sig. (2-tailed)	0.488	0.765	0.668	0.088	0.516	0.088	0.332	0.099	0.932
	*N*	9	9	9	9	9	9	9	9	9
miR-30c	Correlation coefficient	−0.150	−0.033	−0.117	0.567	−0.250	0.483	0.283	−0.500	−0.067
	Sig. (2-tailed)	0.700	0.932	0.765	0.112	0.516	0.187	0.460	0.170	0.865
	*N*	9	9	9	9	9	9	9	9	9
miR-181a	Correlation coefficient	−0.450	−0.267	−0.283	0.550	−0.367	0.667*	0.500	−0.683*	−0.083
	Sig. (2-tailed)	0.224	0.488	0.460	0.125	0.332	**0.050**	0.170	**0.042**	0.831
	*N*	9	9	9	9	9	9	9	9	9
miR-191	Correlation coefficient	−0.033	0.100	0.100	0.483	−0.017	0.350	0.250	−0.383	0.017
	Sig. (2-tailed)	0.932	0.798	0.798	0.187	0.966	0.356	0.516	0.308	0.966
	*N*	9	9	9	9	9	9	9	9	9
miR-196a	Correlation coefficient	−0.467	−0.350	0.017	0.383	0.000	0.183	0.450	−0.233	0.083
	Sig. (2-tailed)	0.205	0.356	0.966	0.308	1.000	0.637	0.224	0.546	0.831
	*N*	9	9	9	9	9	9	9	9	9
STZ group										
miR-16	Correlation coefficient	0.036	0.321	0.000	−0.143	−0.286	0.107	0.393	0.107	0.357
	Sig. (2-tailed)	0.939	0.482	1.000	0.760	0.535	0.819	0.383	0.819	0.432
	*N*	7	7	7	7	7	7	7	7	7
miR-30c	Correlation coefficient	−0.286	0.107	−0.286	−0.036	−0.286	0.464	0.500	−0.250	0.393
	Sig. (2-tailed)	0.535	0.819	0.535	0.939	0.535	0.294	0.253	0.589	0.383
	*N*	7	7	7	7	7	7	7	7	7
miR-181a	Correlation coefficient	−0.107	0.286	0.143	0.107	−0.071	0.036	0.143	0.179	0.107
	Sig. (2-tailed)	0.819	0.535	0.760	0.819	0.879	0.939	0.760	0.702	0.819
	*N*	7	7	7	7	7	7	7	7	7
miR-191	Correlation coefficient	0.036	0.321	0.143	0.429	0.214	0.036	0.036	0.179	0.000
	Sig. (2-tailed)	0.939	0.482	0.760	0.337	0.645	0.939	0.939	0.702	1.000
	*N*	7	7	7	7	7	7	7	7	7
miR-196a	Correlation coefficient	−0.107	−0.250	−0.679	−0.571	−0.821*	0.607	0.857*	−0.571	0.500
	Sig. (2-tailed)	0.819	0.589	0.094	0.180	**0.023**	0.148	**0.014**	0.180	0.253
	*N*	7	7	7	7	7	7	7	7	7
STZ+ICSS group										
miR-16	Correlation coefficient	−0.287	0.310	0.167	−0.310	−0.190	0.524	−0.096	−0.476	0.357
	Sig. (2-tailed)	0.490	0.456	0.693	0.456	0.651	0.183	0.821	0.233	0.385
	*N*	8	8	8	8	8	8	8	8	8
miR-30c	Correlation coefficient	−0.156	0.452	0.214	−0.238	−0.143	0.429	−0.156	−0.357	0.214
	Sig. (2-tailed)	0.713	0.260	0.610	0.570	0.736	0.289	0.713	0.385	0.610
	*N*	8	8	8	8	8	8	8	8	8
miR-181a	Correlation coefficient	−0.443	0.238	0.000	−0.452	−0.357	0.667	0.072	−0.667	0.595
	Sig. (2-tailed)	0.272	0.570	1.000	0.260	0.385	0.071	0.866	0.071	0.120
	*N*	8	8	8	8	8	8	8	8	8
miR-191	Correlation coefficient	−0.216	0.452	−0.024	−0.238	−0.095	0.476	−0.036	−0.429	0.214
	Sig. (2-tailed)	0.608	0.260	0.955	0.570	0.823	0.233	0.933	0.289	0.610
	*N*	8	8	8	8	8	8	8	8	8
miR-196a	Correlation coefficient	−0.024	0.286	−0.429	−0.095	−0.119	0.476	0.419	−0.405	0.024
	Sig. (2-tailed)	0.955	0.493	0.289	0.823	0.779	0.233	0.301	0.320	0.955
	*N*	8	8	8	8	8	8	8	8	8

A: Acquisition session (A1-A5); TQ: Target quadrant; TA: Target annulus; MDT: Mean distance to target; WE: Whishaw’s error. **Correlation is significant at the 0.01 level (2-tailed). *Correlation is significant at the 0.05 level (2-tailed). The table includes all the correlations found in Control, STZ and STZ+ICSS groups according to Spearman’s correlation test (*p* < 0.05, significant correlations are highlighted in the table). Significant correlations have been highlighted in bold and grey box in the table.

**Table 3:** Correlation analyses between miR-16, miR-30c, miR-181a, miR-191 and miR-196a serum levels at 136 days after STZ injection and behavioral variables in Morris Water Maze 2.

miRNA	Ser 2	A1	A2	A3	A4	A5	TQ	TA	MDT	WE
Control group										
miR-16	Correlation coefficient	−0.433	−0.100	−0.267	−0.250	−0.217	0.550	0.617	−0.583	0.417
	Sig. (2-tailed)	0.244	0.798	0.488	0.516	0.576	0.125	0.077	0.099	0.265
	*N*	9	9	9	9	9	9	9	9	9
miR-30c	Correlation coefficient	−0.400	−0.083	−0.383	−0.183	−0.433	0.533	0.467	−0.517	0.483
	Sig. (2-tailed)	0.286	0.831	0.308	0.637	0.244	0.139	0.205	0.154	0.187
	*N*	9	9	9	9	9	9	9	9	9
miR-181a	Correlation coefficient	0.233	0.383	0.183	−0.283	0.133	−0.117	−0.067	0.200	0.783*
	Sig. (2-tailed)	0.546	0.308	0.637	0.460	0.732	0.765	0.865	0.606	**0.013**
	*N*	9	9	9	9	9	9	9	9	9
miR-191	Correlation coefficient	−0.400	−0.083	−0.367	−0.33	−0.350	0.533	0.533	−0.550	0.433
	Sig. (2-tailed)	0.286	0.831	0.332	0.381	0.356	0.139	0.139	0.125	0.244
	*N*	9	9	9	9	9	9	9	9	9
miR-196a	Correlation coefficient	−0.450	−0.350	−0.517	−0.233	−0.433	0.517	0.433	−0.383	0.467
	Sig. (2-tailed)	0.224	0.356	0.154	0.546	0.244	0.154	0.244	0.308	0.205
	*N*	9	9	9	9	9	9	9	9	9
STZ group										
miR-16	Correlation coefficient	−0.214	−0.036	−0.464	−0.679	−0.821*	0.500	0.714	−0.429	0.714
	Sig. (2-tailed)	0.645	0.939	0.294	0.094	**0.023**	0.253	0.071	0.337	0.071
	*N*	7	7	7	7	7	7	7	7	7
miR-30c	Correlation coefficient	−0.214	−0.036	−0.464	−0.679	−0.821*	0.500	0.714	−0.429	0.714
	Sig. (2-tailed)	0.645	0.939	0.294	0.094	**0.023**	0.253	0.071	0.337	0.071
	*N*	7	7	7	7	7	7	7	7	7
miR-181a	Correlation coefficient	−0.179	0.036	−0.357	−0.643	−0.750	0.393	0.679	−0.286	0.643
	Sig. (2-tailed)	0.702	0.939	0.432	0.119	0.052	0.383	0.094	0.535	0.119
	*N*	7	7	7	7	7	7	7	7	7
miR-191	Correlation coefficient	0.107	0.214	−0.500	−0.321	−0.500	0.571	0.679	−0.464	0.929**
	Sig. (2-tailed)	0.819	0.645	0.253	0.482	0.253	0.180	0.094	0.294	**0.003**
	*N*	7	7	7	7	7	7	7	7	7
miR-196a	Correlation coefficient	−0.214	−0.214	−0.714	−0.714	−0.893**	0.679	0.893**	−0.643	0.714
	Sig. (2-tailed)	0.645	0.645	0.071	0.071	**0.007**	0.094	**0.007**	0.119	0.071
	*N*	7	7	7	7	7	7	7	7	7
STZ+ICSS group										
miR-16	Correlation coefficient	0.287	0.333	0.429	−0.238	−0.524	0.119	0.000	0.024	−0.071
	Sig. (2-tailed)	0.490	0.420	0.289	0.570	0.183	0.779	1.000	0.955	0.867
	*N*	8	8	8	8	8	8	8	8	8
miR-30c	Correlation coefficient	0.311	0.381	0.333	−0.167	−0.476	0.190	0.084	−0.048	−0.048
	Sig. (2-tailed)	0.453	0.352	0.420	0.693	0.233	0.651	0.844	0.911	0.911
	*N*	8	8	8	8	8	8	8	8	8
miR-181a	Correlation coefficient	0.108	−0.095	0.333	−0.333	−0.429	−0.214	−0.024	0.333	−0.262
	Sig. (2-tailed)	0.799	0.823	0.420	0.420	0.289	0.610	0.955	0.420	0.531
	*N*	8	8	8	8	8	8	8	8	8
miR-191	Correlation coefficient	0.299	0.238	0.381	−0.214	−0.452	−0.048	0.072	0.238	−0.262
	Sig. (2-tailed)	0.471	0.570	0.352	0.610	0.260	0.911	0.866	0.570	0.531
	*N*	8	8	8	8	8	8	8	8	8
miR-196a	Correlation coefficient	0.263	−0.571	−0.048	0.524	0.452	−0.238	−0.204	0.071	−0.167
	Sig. (2-tailed)	0.528	0.139	0.911	0.183	0.260	0.570	0.629	0.867	0.693
	*N*	8	8	8	8	8	8	8	8	8

A: Acquisition session (A1-A5); TQ: Target quadrant; TA: Target annulus; MDT: Mean distance to target; WE: Whishaw’s Error. **Correlation is significant at the 0.01 level (2-tailed). *Correlation is significant at the 0.05 level (2-tailed). The table includes all the correlations found for Control, STZ and STZ+ICSS groups according to Spearman’s correlation test (*p* < 0.05, highlighted in the table). Significant correlations have been highlighted in bold and grey box in the table.

### The 5-month time-course of the miR-495 and miR-146a serum levels differed in MFB-ICSS treated vs. untreated STZ rats

**Figure 6:**
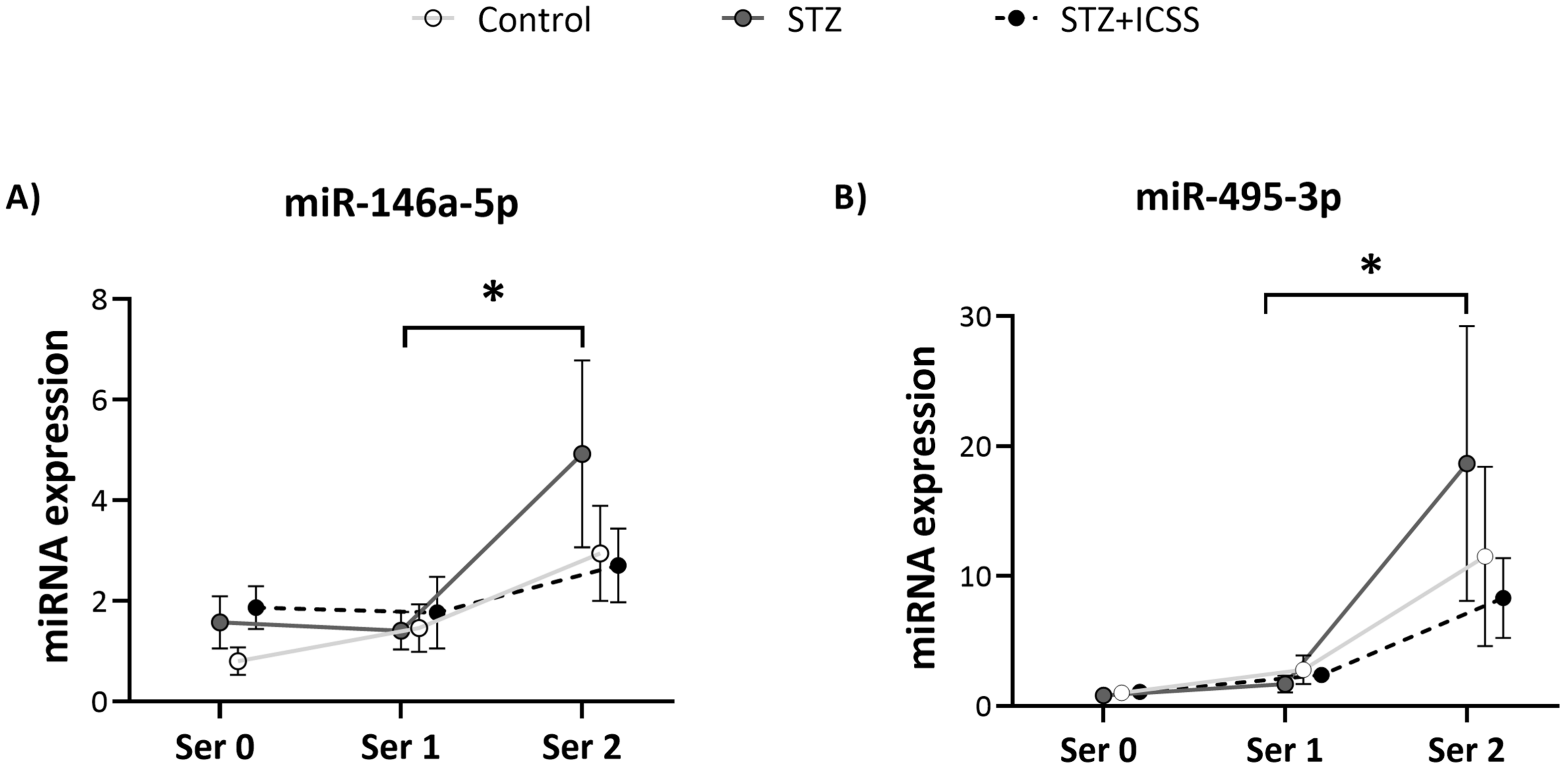
Effects of MFB-ICSS treatment on the longitudinal evolution of miR-146a and miR-495 expression serum levels at 29 (Ser0), 73 (Ser1), and 136 (Ser2) days after STZ injection. Relative quantity of miR-146a **(A)** and miR-495 **(B)** was determined as ^2^(−ΔΔCt) (ΔΔCт = ΔCt sample − ΔCt reference sample; ΔCt = Ct target − Ct normalizer), using the mean in the Control group at Ser0 as the reference sample and miR-let-7a-5p as normalizer. Data are presented as mean ± SEM. *p < 0.05 relative to the Ser2 to Ser3 in the STZ group.

The analysis of serum miRNA expression levels across the three time points (Ser0, Ser1 and Ser2) showed that the 5-month time course of miR-146a and miR-495 were significantly different between the experimental groups (*F*_1,391_ = 6.09, *p* = 0.01; *F*_1,137_ = 5.88, *p* = 0.02, respectively) (Figure 6). Specifically, miR-146a and miR-495 serum levels increased from Ser1 to Ser2 in the STZ group (*p* = 0.03 and *p* = 0.04 respectively), an increase not observed in the STZ+ICCS and Control groups.

### Overexpression of miRNA-495 downregulated mRNA and protein NRF2 levels in human neuroblastoma SH-SY5Y cells

**Figure 7:**
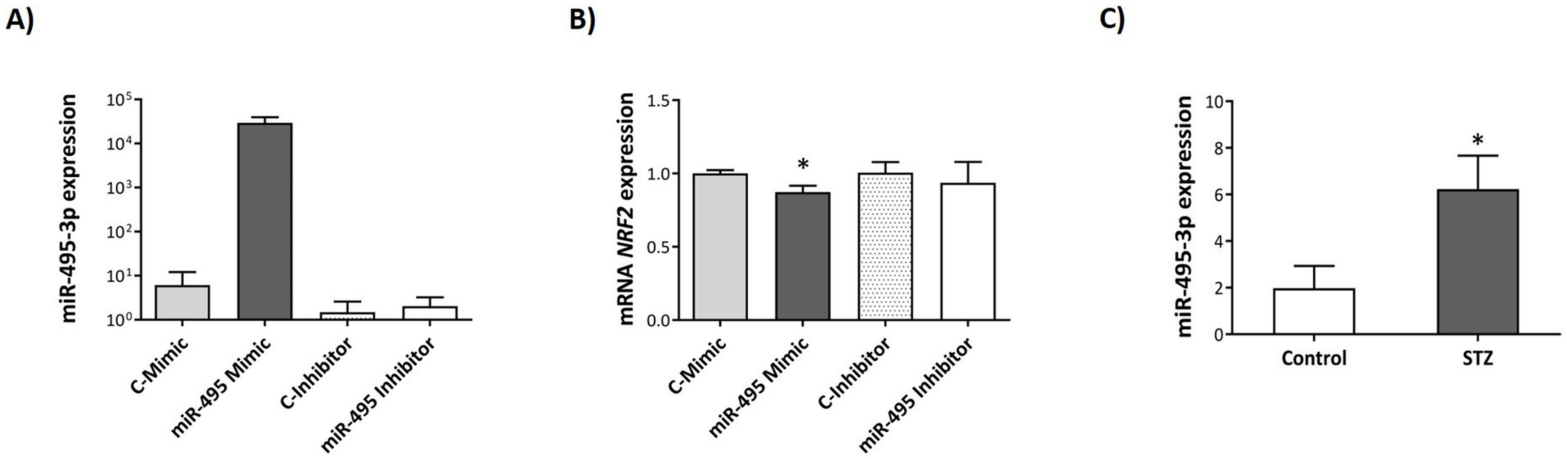
Modification of miR-495 and *NRF2* mRNA expression levels after miR-495 transfection or STZ administration in human neuroblastoma SH-SY5Y cells. Relative expression levels of miR-495 **(A)**, and *NRF2* mRNA **(B)** in SH-SY5Y cells transfected with miR-495 mimic or inhibitor. Relative expression levels of miR-495 **(C)** in SH-SY5Y cells treated with STZ. Relative expression levels were determined as ^2^(−ΔΔCt) (ΔΔCт = ΔCt sample − ΔCt reference sample; ΔCt = Ct target − Ct normalizer). The Control group of each condition was used as the reference sample and miR-let-7a-5p and *HPRT* as normalizer for miRNA and mRNA expression, respectively. Data are presented as mean ± SEM. *p < 0.05 relative to the Control group in each case.

To further determine the potential gene targets of miR-495, transfection assays were performed in SH-SY5Y cells. Transfection efficiency was confirmed by a robust increase in miR-495 expression in cells transfected with the miR-495 mimic (Figure 7A).

**Figure 8:**
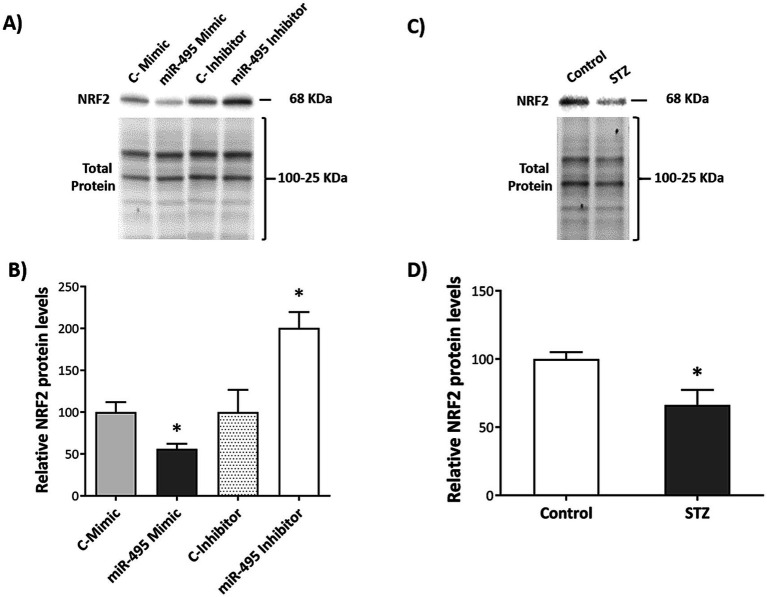
Effect of miR-495 transfection or STZ administration on NRF2 protein levels in human neuroblastoma SH-SY5Y cells. Representative Western Blot results for NRF2 protein, showing total protein band patterns in each case, for SH-SY5Y cells transfected with miR-495 mimic or inhibitor **(A)** or treated with STZ **(C)**. Relative NRF2 protein levels in SH-SY5Y cells transfected with miR-495 mimic or inhibitor **(B)** or treated with STZ **(D)**. The Control group of each condition was used as the reference sample and total protein as normalizer for relative NRF2 protein expression. Data are presented as mean ± SEM. *p < 0.05 relative to the Control group in each case.

Based on the bioinformatic analysis data from the TargetScanHuman (McGeary et al., 2019) and miRDB target (Chen and Wang, 2020) prediction algorithms, 4 candidate target genes of miR-495, *ELOVL7*, *IGF1R*, *TNFRSF1B* and *NRF2*, were selected for experimental validation due to their previously reported involvement in pathways related to the AD pathophysiology. Transfection with either miR-495 mimic or inhibitor did not alter mRNA levels of *ELOVL7*, *IGF1R* and *TNFRSF1B*. Nonetheless, mRNA levels of *NRF2* were significantly reduced in cells transfected with miR-495 mimic (*t_4_* = 2.58, *p* = 0.03, *d* = 2.10) compared to control (Figure 7B). Levels of NRF2 protein were also significantly downregulated following miR-495 mimic transfection (*t_4_* = 3.30, *p* = 0.03, *d* = 2.70), and upregulated after miR-495 inhibitor transfection (*t_4_* = −3.07, *p* = 0.04, *d* = −2.50) in SH-SY5Y cells (Figures 8A,B).

To analyze the effects of STZ administration on miR-495 and NRF2 expression, SH-SY5Y cells were exposed to STZ and compared to vehicle-exposed controls. STZ-exposed cells presented a marked increase in miR-495 expression (*t_8.593_* = −3.534, *p* = 0.007, *g* = 1.43) (Figure 7C), accompanied by a significant downregulation of NRF2 protein levels (*t_6,922_* = 2.746, *p* = 0.03, *g* = 1.42) (Figures 8C,D).

### MFB-ICSS modified NRF2 levels in the hippocampal CA1 region in treated STZ rats

**Figure 9:**
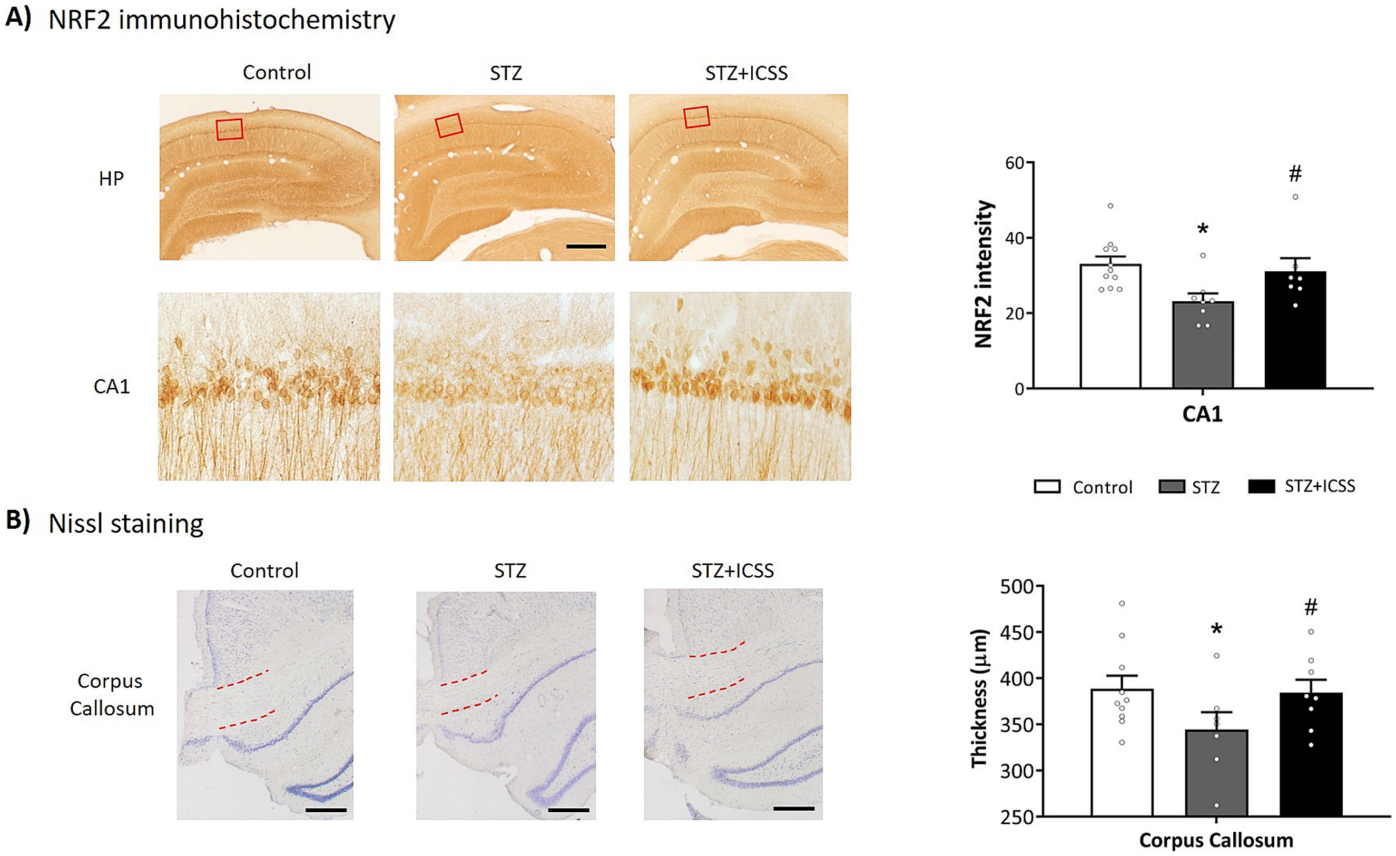
NRF2 levels in the hippocampal CA1 region and corpus callosum thickness after MFB-ICSS treatment. **(A)** Representative photomicrographs showing NRF2 immunostaining in the hippocampus, providing enlarged images of the analyzed CA1 region (red squares) in each experimental group (scale bar = 500 and 50 μm, respectively), and NRF2 intensity levels in the CA1 pyramidal layer. **(B)** Representative photomicrographs for Nissl staining and thickness of corpus callosum. The red lines indicate the measured region. Scale bar = 200 μm. Data are presented as mean ± SEM. *p < 0.05 relative to the Control group and # *p* < 0.05 relative to the STZ group.

Immunohistochemical analysis of NRF2 expression in the CA1 region of the hippocampus revealed significant differences between the three experimental groups (H_2_ = 10.11, ε^2^ = 0.35, *p* = 0.006). In STZ animals, NRF2 levels were downregulated compared to the Control group (*p* = 0.002). Notably, STZ+ICSS rats exhibited significantly higher NRF2 levels than STZ rats (*p* = 0.045) (Figure 9A).

Correlation analysis between NRF2 levels in CA1 and serum miRNAs levels at 5 months post-STZ injection showed a positive correlation with miR-495 (*ρ* = 0.83, *p* = 0.02) within the STZ group, with no significant correlations observed for the other serum miRNAs.

### MFB-ICSS treated STZ rats exhibited increased thickness of corpus callosum compared to untreated STZ rats

Nissl staining showed a significant reduction in corpus callosum thickness in STZ rats compared to the Control group (*t_15_* = 1.88, *p* = 0.04, *g* = 0.88). Within the STZ group, corpus callosum thickness correlated positively with TQ (ρ = 0.83, *p* = 0.04) and negatively with MDT (ρ = −0.83, *p* = 0.04). STZ rats treated with MFB-ICSS showed a significant increase in corpus callosum thickness compared to untreated STZ rats (*t_13_* = 1.71, *p* = 0.05, *g* = 0.83) (Figure 9B).

## Discussion

This study investigated the effects of a prolonged MFB-ICSS treatment on spatial learning and memory, as well as the longitudinal evolution of the serum expression levels of a subset of miRNAs described as potential biomarkers of AD. The study was conducted in a sporadic male rat AD model induced by i.c.v. STZ administration. It also evaluated putative targets of miR-495 involved in several pathways associated with AD pathophysiology, as well as the effects of MFB-ICSS treatment on hippocampal CA1 levels of its target, NRF2, and on corpus callosum thickness, both of which were significantly reduced in untreated STZ rats.

To date, long-term cognitive benefits of DBS remain unclear, and it is critical to determine the stimulation parameters and treatment protocol associated with neuroprotective properties (McKinnon et al., 2019). The vast majority of animal AD studies have employed DBS paradigms lasting no longer than 1 month (Mann et al., 2018; Huang et al., 2019). Recent clinical studies have suggested that alternating DBS at weekly intervals could reduce habituation to the treatment (Seier et al., 2018; Peters and Tisch, 2021). In our study, we applied a prolonged treatment modality consisting of an initial intensive treatment phase followed by spaced administrations over time during a maintenance phase, similar to transmagnetic stimulation protocols (Weiler et al., 2020). Our results show that this MFB-ICSS treatment effectively reversed spatial learning deficits in male STZ rats, as assessed in the MWM, 5 months post-STZ administration.

Consistent with previous studies (Knezovic et al., 2015; Grieb, 2016), untreated STZ rats exhibited performance deficits in the spatial task 1 month post-injection, which persisted until 5 months post-injection. It is indicated by the increased latency values during both MWM1 and MWM2 acquisition sessions. Notably, the impact of the SAD model on spatial learning is severe, as STZ-sham animals showed no significant reduction in latencies across acquisition sessions, either 1 month or 5 months after STZ administration, indicating no training improvement. The STZ-SAD model appears to specifically affect cognitive spatial learning abilities rather than motivational/emotional or sensorimotor functions, as variables like swimming speed and performance in the cued protocol–normal visible-platform training–were unaffected (Hooge and De Deyn, 2001). Deficits would appear to stem from difficulties in adopting effective allocentric search strategies essential for spatial navigation.

Although the SAD model causes severe and long-lasting cognitive impairments in the spatial task in the MWM, MFB-ICSS alleviated these deficits, aligning the performance of the treated STZ animals with that of the controls. The rescue effect attributed to ICSS treatment is evident in the spatial tests conducted at 5 months, following the completion of the stimulation protocol. In MWM2, the STZ+ICSS animals, compared to the STZ group, showed significantly better performance during the acquisition sessions and remembered the platform location after 72 h. Future follow-up studies are required to assess whether the effects of this prolonged MFB-ICSS treatment will remain in the long-term.

Moreover, STZ rats receiving MFB-ICSS changed from predominantly semi-spatial strategies in MWM1 to spatial strategies in MWM2 compared to untreated STZ rats. These findings indicate that ICSS may exert beneficial effects beyond simply improving escape latency, potentially enhancing the cognitive strategies used to navigate the task and locate the hidden platform. Our results suggest that MFB stimulation could be a promising therapeutic target for SAD. MFB stimulation physiologically activates memory-related areas, as well as neuroanatomical systems associated with functions that could play an important adjunctive role in AD patients, such as reinforcement, motivation and emotions (Shirvalkar et al., 2006; Aldavert-Vera et al., 2013; Kádár et al., 2016). ICSS at the MFB may induce upstream and downstream effects in related neural circuits by targeting key nodes within the AD brain’s neural network, enhancing connectivity and thereby alleviating AD symptoms. Furthermore, the unilateral administration of ICSS in this protocol aligns with trends toward using less invasive DBS (unilateral, among other aspects) in other targets, such as the fornix or the basal nucleus of Meynert (Lv et al., 2018). Future DBS studies in validated animal models will be critical for guiding the selection of neuroanatomical targets and stimulation parameters associated with optimal neuroprotective properties.

This study also conducted a longitudinal analysis of serum expression levels of selected miRNAs, previously described as synaptic plasticity regulators and postulated as potential diagnostic biomarkers of AD. Serum levels of miR-16, miR-30c, miR-181a, miR-191, and miR-196a were significantly upregulated in AD rats 29 days after STZ administration, and these levels correlated with spatial task performance 4 months later. miR-30c, miR-181a and miR-196a have been shown to be upregulated in the blood of AD patients (Kumar and Reddy, 2016; Islam et al., 2021; Besli et al., 2023). However, miR-16 and miR-191 were found to be downregulated in the white matter, cortex and/or serum of AD patients (Dong et al., 2015; Sun et al., 2022). Overexpression of miR-16 reduces APP protein expression in the hippocampus of senescence accelerated (SAMP8) mice, and consequently, low expression of miR-16 may lead to APP protein accumulation in an early-onset AD animal model (Liu et al., 2012). In this context, an increase in miR-16 levels could reflect a neuroprotective response aimed at reducing abnormal APP accumulation in the brains of STZ rats, which are potentially in an early to mid-stage of SAD at 29 days post-injection (Knezovic et al., 2015). Similarly, miR-191 attenuates Tau phosphorylation, amyloidogenic processing of APP and neuronal cell death, suggesting that higher levels of miR-191 may protect neurons from neuropathological changes in AD (Wang et al., 2022). Given the multiple processes that might be regulated by a single miRNA, further studies are needed to elucidate their mechanisms of action to better understand their roles in AD pathogenesis.

Regarding changes in miRNA levels during disease progression, a significant upregulation of baseline blood levels of miR-181a in patients with mild cognitive impairment who converted to AD has been reported (Islam et al., 2021). They proposed that miR-181a, along with miR-146a and miR-148a in a three-miRNA signature, regulates cellular processes essential for cognitive function. Our findings suggest that miR-16, miR-30c, miR-191 and miR-196a may also serve as molecular markers indicative of cognitive status, potentially predicting cognitive decline in AD. Further longitudinal follow-up studies are required to confirm this hypothesis and investigate whether the initially elevated levels of these miRNAs remain significantly altered in late-stage AD.

Since the sporadic AD rat model based on the STZ injection recapitulates some of the plasma miRNA expression changes observed in AD patients, we proposed its use as a suitable model for studying the effects of the MFB-ICSS treatment on the temporal evolution of circulating miRNAs levels. For the first time, we show that a DBS treatment modulates the serum expression patterns of miR-146a and miR-495 during AD progression, suggesting that both miRNAs may be influenced by the MFB-ICSS treatment. miR-146a plays a key role in immune response and it is upregulated by reactive oxygen species (Nunomura and Perry, 2020). Many studies have analyzed its levels in AD patients, although the results have been divergent. Consistent with our findings, one study reported no alterations in plasma miR-146a levels in AD patients but did find correlations with illness severity (Maffioletti et al., 2020). Regarding miR-495, its specific role in AD remains understudied. Wang et al. reported its downregulation in the cortex of AD patients (Wang et al., 2011). No studies to date have evaluated its circulating levels in either AD animal models or clinical populations. Here, we assessed circulating miR-495 levels in an animal model of SAD and performed transfection assays using a miR-495 mimic and inhibitor in the human SH-SY5S cell line to validate predicted targets related to AD pathophysiology. We selected four candidates: ELOVL7, involved in lipid dyshomeostasis (Peña-Bautista et al., 2021); IGF1R linked to neuroinflammation and Aβ accumulation (Sohrabi et al., 2020); TNFRSF1B, associated with cerebrospinal fluid t-tau and p-tau levels (Pillai et al., 2021) and NRF2, a key regulator of antioxidant response (Rana et al., 2025). Our results confirmed NRF2 as a target of miR-495. Although miR-495 has previously been shown to interact with the NRF2-ARE pathway in the course of epileptogenesis (Geng et al., 2018), no prior studies have explored this interaction in the AD context. Using SH-SY5Y cells treated with STZ, considered an appropriate *in vitro* AD model (Guo et al., 2017; Ranjan et al., 2018; Cheng et al., 2021), we demonstrated that STZ exposure led to increased levels of miR-495 and decreased levels of its target, NRF2, in the context of a human in vitro model of AD. This is the first study showing that STZ administration affects miRNA expression in human SH-SY5Y cells.

Interestingly, in the animal SAD model, serum miR-495 levels were positively correlated with NRF2 protein levels in the hippocampal CA1 region. Moreover, MFB-ICSS treatment reversed the downregulation of NRF2 observed in STZ rats. These findings suggest that miR-495 could be an essential miRNA orchestrating the mechanisms of MFB-ICSS memory improvements, potentially offering a novel therapeutic target for alleviating AD pathology. Further studies are required to examine the potential therapeutic effect of miR-495 *in vivo*.

Finally, MFB-ICSS treatment reversed corpus callosum degeneration observed in untreated STZ rats. Decreased thickness of corpus callosum (Voronkov et al., 2019) and reduced fractional anisotropy values indicating microstructural abnormalities of this area (Tristão Pereira et al., 2021), have been reported in STZ rats. Recent studies suggest that white matter degeneration and demyelination abnormalities may play a mechanistic role in AD pathology and could serve as potential therapeutic targets (Nasrabady et al., 2018). Approximately 80% of the dry weight of myelin is composed of lipids (O’Brien and Sampson, 1964) and oxidative stress in the brain is largely manifested through lipid peroxidation (Yin, 2023). Therefore, treatments targeting antioxidant proteins, such as NRF2, may help to prevent or repair myelin damage by alleviating the lipid-related oxidative changes. Several studies have also shown that DBS treatments can modulate white matter integrity in various neurological disorders (Cattarinussi et al., 2022; Huang et al., 2022). Thus, further research should evaluate the effects of DBS on myelin integrity and oxidative stress, both of which may contribute to the neurotoxic environment associated with disease progression characteristic of AD (Yin, 2023).

In conclusion, a prolonged MFB-ICSS treatment, which mitigated cognitive difficulties in a spatial task observed in a SAD rat model induced by STZ administration, modulated circulating levels of miRNA-495 and miR-146a. Mechanistically, we identified the antioxidant protein NRF2 as a target of miR-495 and demonstrated that MFB-ICSS treatment increased NRF2 expression in the hippocampal CA1 region, as well as of corpus callosum thickness, both of which were significantly reduced in untreated STZ rats.

## Data Availability

The raw data supporting the conclusions of this article will be made available by the authors, without undue reservation.

## Supplementary material

SUPPLEMENTARY FIGURE S1 Effect of STZ on miRNAs expression serum levels in rats 73 (Ser1) and 136 (Ser2) days after STZ injection. Relative quantity of each target miRNA in Ser1 (A) and Ser2 (B) was determined as ^2^(-ΔΔCt) (ΔΔCт = ΔCt sample − ΔCt reference sample; ΔCt = Ct target − Ct normalizer), using the mean level of Ser0 in the Control group as the reference sample and miR-let-7a-5p as normalizer. Data are presented as mean ± SEM.
