# Supplementary figures and images for: Intracranial self-stimulation mitigates spatial task deficits, modifies miR-146a and miR-495 serum levels and restores hippocampal NRF2 levels in a rat model of sporadic Alzheimer’s disease
Source: Front Aging Neurosci. 2025 Nov 11;17:1671196. doi: 10.3389/fnagi.2025.1671196 (PMC12644048; doi:10.3389/fnagi.2025.1671196)

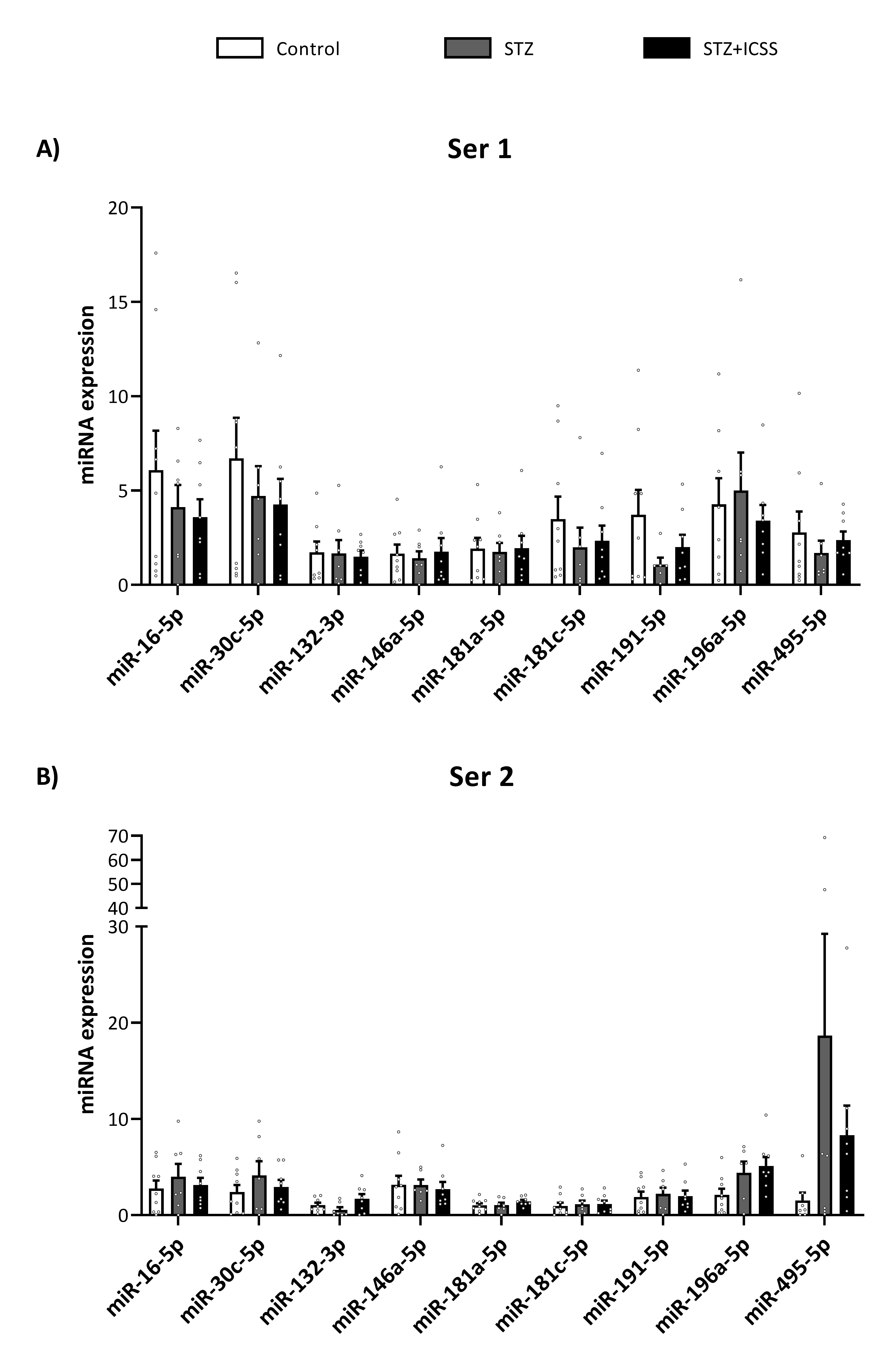

Supplement: SUPPLEMENTARY FIGURE S1 — Effect of STZ on miRNAs expression serum levels in rats 73 (Ser1) and 136 (Ser2) days after STZ injection. Relative quantity of each target miRNA in Ser1 (A) and Ser2 (B) was determined as 2(-ΔΔCt) (ΔΔCт = ΔCt sample − ΔCt reference sample; ΔCt = Ct target − Ct normalizer), using the mean level of Ser0 in the Control group as the reference sample and miR-let-7a-5p as normalizer. Data are presented as mean ± SEM. [file Image_1.TIF]
